# Patient-derived monoclonal antibodies to SARS-CoV-2 nucleocapsid protein N-terminal and C-terminal domains cross-react with their counterparts of SARS-CoV, but not other human betacoronaviruses

**DOI:** 10.3389/fimmu.2023.1093709

**Published:** 2023-01-31

**Authors:** Yingfen Wen, Wenjing Guo, Yuyi Min, Kexin Zhong, Xulei Zhang, Xiaomin Xing, Yuwei Tong, Yuejun Pan, Wenxin Hong, Weiping Cai, Lei Yu

**Affiliations:** Guangzhou Eighth People’s Hospital, Guangzhou Medical University, Guangzhou, China

**Keywords:** SARS-CoV-2, coronavirus, COVID-19, nucleocapsid protein, N-terminal, C-terminal, monoclonal antibody, cross-reaction

## Abstract

**Introduction:**

SARS-CoV-2 nucleocapsid (N) protein plays a key role in multiple stages of the viral life cycle such as viral replication and assembly. This protein is more conserved than the Spike protein of the virus and can induce both humoral and cell-mediated immune responses, thereby becoming a target for clinical diagnosis and vaccine development. However, the immunogenic characteristics of this protein during natural infection are still not completely understood.

**Methods:**

Patient-derived monoclonal antibodies (mAbs) against SARS-CoV-2 N protein were generated from memory B cells in the PBMCs using the antigen-specific B cell approach. For epitope mapping of the isolated hmAbs, a panel of series-truncated N proteins were used , which covered the N-terminal domain (NTD, aa 46-174 ) and C-terminal domain (CTD, aa 245-364 ), as well as the flanking regions of NTD and CTD. NTD- or CTD-specific Abs in the plasma from COVID-19 patients were also tested by ELISA method. Cross-binding of hmAbs or plasma Abs in COVID-19 patients to other human β-CoV N proteins was determined using the capture ELISA.

**Results:**

We isolated five N-specific monoclonal antibodies (mAbs) from memory B cells in the peripheral blood of two convalescent COVID-19 patients. Epitope mapping revealed that three of the patient-derived mAbs (N3, N5 and N31) targeted the C-terminal domain (CTD), whereas two of the mAbs (N83 and 3B7) targeted the N-terminal domain (NTD) of SARS-CoV-2 N protein. All five patient-derived mAbs were cross-reactive to the N protein of SARS-CoV but showed little to no cross-reactivity to the N proteins of other human beta coronaviruses (β-CoVs). We also tested 52 plasma samples collected from convalescent COVID-19 patients for Abs against the N proteins of human β-CoVs and found that 78.8% of plasma samples showed detectable Abs against the N proteins of SARS-CoV-2 and SARS-CoV. No plasma sample had cross-reactive Abs to the N protein of MERS-CoV. Cross-reactive Abs to the N proteins of OC43 and HKU1 were detected in 36.5% (19/52) and 19.2% (10/52) of plasma samples, respectively.

**Discussion:**

These results suggest that natural SARS-CoV-2 infection elicits cross-reactive Abs to the N protein of SARS-CoV and that the five patient-derived mAbs to SARS-CoV-2 N protein NTD and CTD cross-react with their counterparts of SARS-CoV, but not other human β-CoVs. Thus, these five patient-derived mAbs can potentially be used for developing the next generation of COVID-19 At-Home Test kits for rapid and specific screening of SARS-CoV-2 infection.

## Introduction

SARS-CoV-2 is the causative agent of the coronavirus disease 2019 (COVID-19) pandemic that has swept across the world as one of the most devastating public health crises. SARS-CoV-2 nucleocapsid (N) protein, one of the four viral structural proteins including spike (S), N, envelope (E), and membrane (M) proteins, is a multifunctional protein comprising of a total of 419 amino acids and plays a critical role in many aspects of the viral life cycle such as viral replication, transcription, and genome packaging (1–3). The N protein has three distinct and highly conserved domains, namely the N-terminal domain (NTD), the disordered central linker region (LKR), and the C-terminal domain (CTD) (4–8). The NTD contains the RNA-binding domain that binds to viral genomic RNA to form the viral ribonucleoprotein core, while the CTD has a dimerization domain that allows the N protein to form dimers and N protein-RNA clusters (4–8). The NTD and CTD functional domains are connected by the LKR that ensures flexibility of the N protein (1). Ultimately, the N protein drives compaction of viral genome RNA and viral proteins for virion assembly (1).

The N protein is the most abundant viral protein that presents early in blood and saliva during asymptomatic and symptomatic SARS-CoV-2 infection (9, 10). The N protein is highly immunogenic and strongly elicits antibody (Ab) responses in COVID-19 patients (2, 11, 12). As a fact, detection of anti-N Abs is more sensitive than detection of anti-S Abs during the early stage of SARS-CoV-2 infection (12–14). In addition, anti-N protein Abs can be used as a marker of vaccine breakthrough infection, as most COVID-19 vaccines listed for emergency use by the World Health Organization (WHO) are S protein-encoding vaccines such as mRNA vaccines, viral vector vaccines, and protein subunit vaccines that can induce neutralizing Abs against S protein, but not anti-N protein Abs due to the absence of the N protein/mRNA/gene in these vaccines. The N protein and its specific Abs have not only been used as primary targets for clinical laboratory diagnosis of COVID-19 in the early phase of infection but also as major components of COVID-19 At-Home Test kits that detect SARS-CoV-2 N protein in self-collected nasal swab specimens.

SARS-CoV-2, along with SARS-CoV and MERS-CoV (Middle East respiratory syndrome coronavirus), belongs to the genus Betacoronavirus (Beta-CoV or β-CoV). These three highly pathogenic β-CoVs cause severe acute respiratory syndrome in humans, leading to large disease outbreaks (15–17). There are two more β-CoVs (OC43 and HKU1) that can infect humans to cause the common cold (18, 19). Among these human β-CoVs, the N proteins are relatively conserved in terms of amino acid sequences, structures, and functions (20). The N protein of SARS-CoV-2 shows approximately 90%, 49%, 39%, and 31% amino acid sequence homology with the N proteins of SARS-CoV (21, 22), MERS (23), OC43 (5), and HKU1 (5), respectively. Since SARS-CoV-2 and SARS-CoV N proteins are highly similar, they likely share more common antigenic epitopes than other human β-CoVs. Indeed, a recent study has demonstrated that animal-derived monoclonal Abs (mAbs) against SARS-CoV structural proteins, including N, S, and M proteins, can strongly cross-react with their protein counterparts of SARS-CoV-2 (24). In addition, sera from SARS-CoV patients can react with SARS-CoV-2 N and S proteins (25). Furthermore, pre-pandemic serum samples from individuals who were never infected with SARS-CoV have cross-reactive IgG Abs against SARS-CoV-2 (26, 27), indicating that cross-reactive IgG Abs are likely elicited by other human CoVs such as those responsible for the common cold. Similarly, convalescent COVID-19 sera contain high levels of cross-reactive Abs to SARS-CoV structural proteins (28–30), indicating robust cross-reactive Abs are elicited during natural SARS-CoV-2 infection. These findings demonstrate that SARS-CoV-2 structural proteins such as the N protein share some common antigenic epitopes with other human CoVs, particularly human β-CoVs, and thereby elicit cross-reactive Ab responses.

Several approaches such as protein microarrays and enzyme immunoassays (EIAs) have been used to profile and characterize antigenic epitopes of the N proteins of SARS-CoV-2 and SARS-CoV (21, 31–36). In these assays, reactivation strengths of synthetic peptides spanning the full-length N proteins with sera from SARS patients or genetically expressed N proteins with animal-derived mAbs are analyzed to determine the immunodominant epitopes (21, 31–36). These approaches represent powerful ways for mapping antigenic determinants or B cell epitopes. In this report, we expanded such studies by using patient-derived mAbs against SARS-CoV-2 N protein. We isolated five N-specific mAbs from memory B cells in the peripheral blood of two individuals who had recovered from COVID-19. Epitope mapping revealed that three of these human mAbs (hmAbs) were reactive with the CTD, while the other two hmAbs targeted the NTD of SARS-CoV-2 N protein. All five hmAbs were cross-reactive to the N protein of SARS-CoV but showed little to no cross-reactivity to the N proteins of other human β-CoVs. We also demonstrate that Abs in convalescent COVID-19 sera against the N protein NTD and CTD are elicited during SARS-CoV-2 natural infection, and cross-react with their counterparts of SARS-CoV. These patient-derived mAbs can be potentially used for developing next generation COVID-19 At-Home Test kits for rapid screening of SARS-CoV-2 infection and EIAs for clinical laboratory diagnosis of COVID-19.

## Materials and methods

### Plasma and peripheral blood mononuclear cell (PBMC) samples

This study was performed with the approval of the Institutional Review Boards (IRB) at the Guangzhou Eighth People’s Hospital (20200134). Blood samples were drawn after each participant provided a written informed consent form. Fifty-two plasma samples were collected from convalescent COVID-19 patients at time points from one week to six weeks post-infection. These patients had positive RNA results for SARS-CoV-2 infection and were hospitalized at Guangzhou Eighth People’s Hospital, Guangzhou Medical University, China. In addition, 18 plasma samples from healthy blood donors were collected before the SARS-CoV-2 pandemic and were used for comparative analyses. Plasma samples were clarified, aliquoted, and stored at -80°C until use. Fresh PBMCs from two COVID-19 patients after four months of SARS-CoV-2 infection were used for B cell isolation using fluorescence-activated cell sorting (FACS). All blood samples were collected before or on May of 2020, the early outbreak of COVID-19 in China.

### Expression of human β-CoV N proteins in mammalian cells

The coding sequence of the full-length N protein of SARS-CoV-2 (NC_045512.2), SARS-CoV (NC_004718.3), MERS-CoV (JX869059.2), OC43 (AY585228.1), or HKU1 (NC_006577.2) was inserted into pcDNA3.1 expression vector (Invitrogen, Waltham, MA) with a D7-tag on the C-terminus of each N protein gene as previously reported (37). The recombinant plasmid was transfected into mammalian 293T cells. After culturing, N protein-containing supernatants were collected and clarified by centrifugation. Aliquots of supernatants were added with a protease inhibitor and then stored at -20°C until use.

### Western blot

Western blot was used for analyzing N protein expression in culture supernatants of transfected 293T cells. Briefly, 20 µl boiled supernatant sample in reducing buffer was loaded into the 10% precast SDS-PAGE gel (Tris-GlyBeyoGel™ Plus PAGE, Beyotime Biotechnology, China) and followed by high-performance electrophoresis. Proteins were blotted onto a polyvinylidene difluoride (PVDF) membrane (Millipore, Billerica, MA). The expression of N protein was probed by the anti-D7 tag Ab (Cliniqa, San Marcos, CA) and the horseradish peroxidase-conjugated secondary Ab (Jackson ImmunoResearch, West Grove, PA). The supernatant collected from the 293T cell culture without transfection or transfected with empty pcDNA3.1 vector was used as a negative control of N protein expression.

### Isolation and cloning of patient-derived mAbs against SARS-CoV-2 N protein

Patient-derived mAbs against SARS-CoV-2 N protein were generated from memory B cells (CD19^+^IgD^-^IgM^-^CD27^+^CD38^low^, **Supplementary Figure 1**) in the PBMCs from COVID-19 patients using the antigen-specific B cell approach as previously reported (38). Briefly, memory B cells in fresh PBMCs from convalescent COVID-19 patients were sorted into 96-well culture plates with 100 cells per well. B cells were cultured for 7-10 days in culture medium supplemented with growth factors and stimuli including IL-21, Chk2 inhibitor, and CpG in the presence of feeder cells (irradiated PBMCs from healthy blood donors). The culture supernatants were screened for N protein binding using a capture ELISA. The ELISA microplates precoated with anti-D7 tag Ab were used to capture the N protein with D7-tag in the supernatant of 293T cells transfected with recombinant pcDNA3.1. Cells in the positive wells were subjected to RNA extraction for cDNA synthesis using the SuperScript™ III First-Strand Synthesis System (Invitrogen, Waltham, MA). The cDNA was used as a template of the nested PCR with gene-specific primers or primer mixes to amplify the transcripts of Ab heavy and light variable genes as previously reported (39). The PCR products were purified from agarose gel using QIAEX II Gel Extraction Kit (Qiagen, Hilden, Germany). Purified PCR products were digested with restriction enzymes AgeI/SalI, AgeI/BsiWI, or AgeI/XhoI (ThermoFisher Scientific, Waltham, MA) and followed by ligation into human IgG1, Igκ, or Igλ expression vectors (39). The recombinant Ab-expressing constructs were sequenced and analyzed using the IMGT/V-QUEST (IMGT, France), a sequence alignment software for the immunoglobulin sequences of the variable regions.

### Affinity measurement and epitope binning

The K_D_ measurement and epitope binning of isolated hmAbs to SARS-CoV-2 N protein were conducted on the Octet K2 System (ForteBio, Fremont, CA) using the Bio-Layer Interferometry (BLI) as previously reported (40). Briefly, the Streptavidin (SA) sensor captured the biotin-labeled (EZ-Link™ NHS-PEG12-Biotin, ThermoFisher Scientific, Waltham, MA) anti-D7 Ab at 80 µg/ml, which subsequently captured the SARS-CoV-2 N protein in the supernatant. Serially diluted Abs against the N protein were added. After washing, the association and dissociation curves were obtained. The K_D_ values were calculated using Data Analysis SPSS 11.0. For epitope binning, the two Abs were added in tandem. Residual binding was calculated as the percent binding of secondary Ab in the presence of primary Ab relative to the binding of secondary Ab alone.

### Epitope mapping

For epitope mapping of the isolated hmAbs, a panel of series-truncated N proteins including N(1-174), N(1-364), N(46-174), N(245-364), and N(245-419), each with D7-tags at the C-terminus, were expressed in mammalian 293T cells. These N subdomains/fragments covered the NTD (46-174) and CTD (245-364), two main domains of the N protein, as well as the flanking regions of NTD and CTD. The binding of each hmAb to these truncated N proteins was measured using a capture ELISA. The N protein fragments and capture ELISA were also used to test NTD- or CTD-specific Abs in the plasma from COVID-19 patients. The area under the curve (AUC) was calculated using GraphPad Prism 8.

### Cross-reactivity analysis

Cross-binding of hmAbs or plasma Abs in COVID-19 patients to other human β-CoV N proteins was determined using the capture ELISA. The N protein of SARS-CoV, MERS-CoV, OC43, or HKU1 was anchored by precoated D7-tag capture Ab. Human mAbs starting at 10 µg/ml were used for preparing a 3-fold serial dilution that was applied to the capture ELISA. To test cross-reactive Abs in plasma samples, each plasma sample at a 1:100 dilution was applied to the capture ELISA. In addition to these COVID-19 patient samples, 18 plasma samples from healthy blood donors collected before the SARS-CoV-2 pandemic were also analyzed. All samples were detected in duplicate. The threshold was calculated by the OD value of negative controls with standard deviation (SD) in each microplate.

### Statistical analysis

Statistical analysis was performed using GraphPad Prism 8.0. Data were expressed as mean ± SD. Differences between 2 groups were compared using unpaired t tests or Mann–Whitney U test. P<0.05 was considered statistically significant.

## Results

### SARS-CoV-2 N-specific mAbs derived from convalescent COVID-19 patients

**Figure 1:**
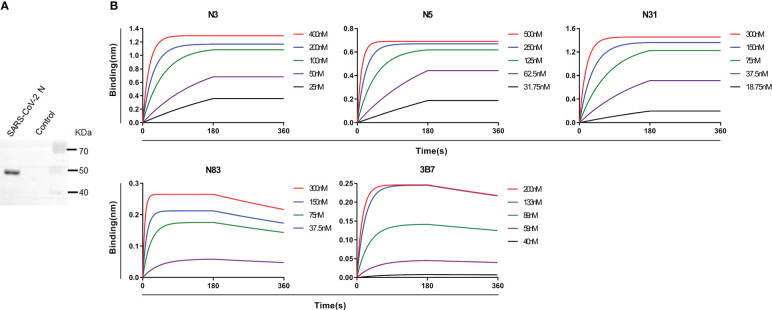
Isolation and characterization of patient–derived mAbs to SARS–CoV–2 N protein. **(A)** Western blot showing SARS–CoV–2 N protein in the supernatant of transfected 293 T cells. Supernatant from 293 T cells transfected with empty pcDNA3.1 vector was used as a control. **(B)** BLI results showing the affinities of 5 patient–derived mAbs to SARS –CoV–2 N protein. The N protein was captured by anti–tag Ab with biotin labeling, which was immobilized on SA sensor. The data of association and disassociation between hmAbs and N protein were acquired on an Octet K2 platform. Experiments were repeated at least 3 times.

To isolate mAbs from memory B cells (CD19^+^IgD^-^IgM^-^CD27^+^CD38^low^) in the peripheral blood of COVID-19 patients, we genetically expressed full-length SARS-CoV-2 N protein in mammalian 293 T cells. As shown in **Figure 1A**, the full-length SARS-CoV-2 N protein was detected in supernatant from 293 T cells transfected with recombinant pcDNA3.1 plasmids with an insert of SARS-CoV-2 N/D7-Tag fusion gene, whereas supernatant from 293 T cells transfected with an empty pcDN3.1 vector did not show any protein band. This genetically expressed full-length SARS-CoV-2 N protein was used to screen Ab-producing memory B cells in B cell culturing plates. B cells in Ab-positive wells were subjected to RNA extraction and subsequently RT-PCR amplification of genes expressing Ab heavy and light chains. As shown in **Table 1**, five mAbs against SARS-CoV-2 N protein, namely N3, N5, N31, N83, and 3B7, were successfully obtained from fresh memory B cells in the peripheral blood of two convalescent COVID-19 patients. Three of these hmAbs (N3, N5, and N31) were isolated from one male adult COVID-19 patient. These three hmAbs had the same VH gene, HV1-69, but had different sequences and lengths in their complementarity-determining region 3 (CDR3). These hmAbs were also paired with different light chains, KV4-1, LV1-51, and KV3-15, respectively (**Table 1**). N3, N5, and N31 hmAbs all exhibited high-affinity binding to SARS-CoV-2 N protein with the K_D_ values below 1 pM (**Figure 1B**; **Table 2**). The two other hmAbs, N83 and 3B7, were obtained from a female COVID-19 patient. N83 had HV4-61 VH gene that was paired with KV2-28 light chain, while 3B7 had VH1-24 VH gene that was paired with KV3-20 light chain. N83 and 3B7 had K_D_ values of 1.76 nM and 1.94 nM, respectively (**Figure 1B**; **Table 2**). Thus, N83 and 3B7 also exhibited high-affinity binding to SARS-CoV-2 N, albeit at lower degrees of reactivation strengths when compared to N3, N5 and N31.

**Table 1:** Gene characteristics of five N–specific mAbs derived from two convalescent COVID–19 patients.

Ab ID	Heavy chain				Light chain			
	V family	CDR3 sequence	CDR3 length	Identity(%)	V family	CDR3 sequence	CDR3 length	Identity(%)
N3	IGHV1–69	CARGGYCSGANCPKWGEWSHSYNYMDVW	28	96.88	IGKV4–1	CQQYYKSPGTF	11	97.31
N5	IGHV1–69	CARGGGGVERGVILTRWFDPW	21	96.53	IGLV1–51	CGTWDNSLSAGVF	13	98.60
N31	IGHV1–69	CARGMWSNPPGYCCSYMDVW	20	96.53	IGKV3–15	CQQYSNWPRTF	11	94.98
N83	IGHV4–61	CARGGMAVGAPLYYYFYGMDVW	22	92.78	IGKV2–28	CMQALQTLSITF	12	97.28
3B7	IGHV1–24	CATPAPTIVGVVIYALHIW	19	93.40	IGKV3–20	CLQYTTSPPLTF	12	91.49

**Table 2:** Affinities of five N–specific hmAbs detected by BLI.

Antibody ID	K_D_ (M)	kon(1/Ms)	koff(1/s)
N3	<1.0E–12	1.57E+05	<1.0E–07
N5	<1.0E–12	1.37E+05	<1.0E–07
N31	<1.0E–12	1.74E+05	<1.0E–07
N83	1.76E–09	6.39E+05	1.13E–03
3B7	1.94E–09	3.55E+05	6.89E–04

### Patient-derived mAbs recognize the functional NTD and CTD domains

**Figure 2:**
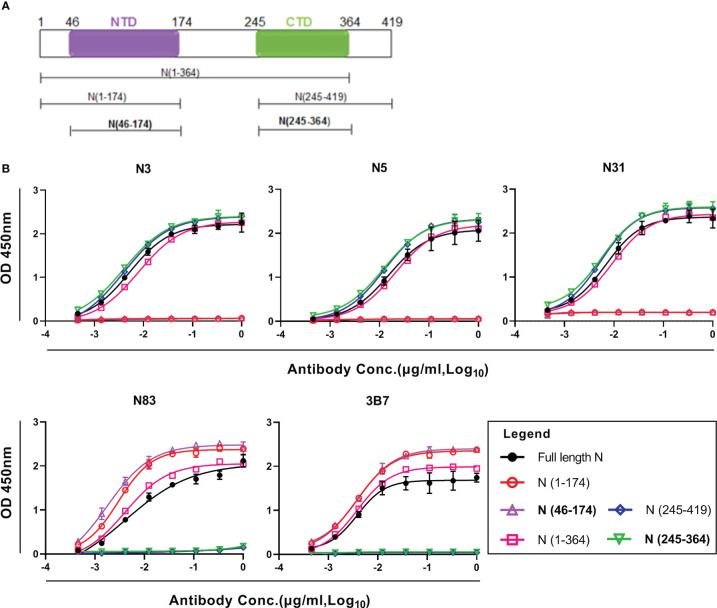
Epitope mapping of patient–derived mAbs to SARS–CoV–2 N protein. **(A)** Schematic of the SARS –CoV–2 N protein structure and domains. **(B)** ELISA results showing the binding curves of 5 hmAbs to full–length and fragments of SARS–CoV–2 N proteins. All samples were detected in duplicate. NTD, N–terminal domain; CTD, C–terminal domain.

To map antigenic epitopes of these five hmAbs to SARS-CoV-2 N protein, a panel of overlapping N protein fragments that covered the entire SARS-CoV-2 N protein sequence were expressed (**Supplementary Figure 2**). These N protein fragments were used for mapping antigenic epitopes of the five hmAbs using ELISA assays. As shown in **Figure 2**, these five hmAbs fell into two categories based on their reactivation strengths. N3, N5, and N31 hmAbs recognized the CTD domain (**Figures 2A, B**), while N83 and 3B7 hmAbs selectively bound to the NTD domain (**Figures 2A, B**). Further analysis of Ab competition by BLI showed that N3, N5, and N31 fully competed with each other, but did not compete with the NTD-specific hmAbs (**Supplementary Figure 3**). These results suggest that three hmAbs against the CTD may have the same or overlapping binding epitopes, but have different binding epitopes with two hmAbs that were responsive to the NTD.

### Patient-derived mAbs cross-react with SARS-CoV N

**Figure 3:**
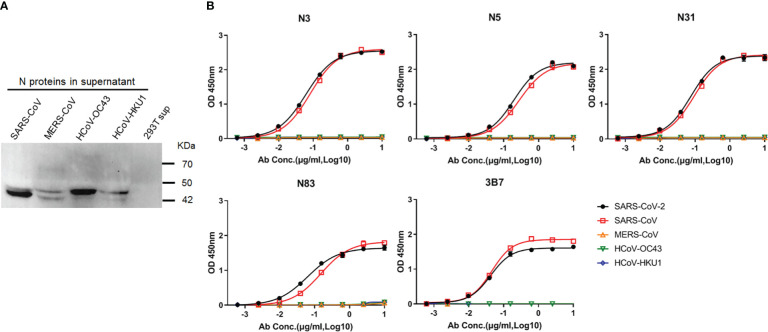
Patient–derived mAbs cross–reacted with the N proteins of human β–CoVs. **(A)** Western blot showing N proteins in the supernatant of 293 T cells that were transfected with recombinant pcDNA3.1 constructs to express N protein of SARS–CoV, MERS–CoV, OC43, or HKU1. Supernatant of 293 T cells transfected with empty pcDNA3.1 vector (293T sup) was used as a negative control. **(B)** ELISA results showing the binding curves of Abs to N proteins of SARS–CoV, MERS–CoV, OC43, and HKU1.

Since SARS-CoV-2 N protein shares substantial sequence conservation with the protein counterparts of other human β-CoVs, particularly SARS-CoV (20–23), these patient-derived mAbs might cross-react with other human β-CoV N proteins. To clarify this speculation, we genetically expressed N proteins of SARS-CoV, MERS-CoV, OC43, and HKU1 (**Figure 3A**). These N proteins were used for testing cross-activities of the five hmAbs. As shown in **Figure 3B**, all five hmAbs were cross-reactive to the N protein of SARS-CoV. None of these five hmAbs were reactive to any of the N proteins from MERS-CoV, OC43, or HKU1 (**Figure 3B**; **Supplementary Figure 4**). Thus, the five patient-derived mAbs to SAR-CoV-2 N protein NTD and CTD cross-react with their N protein counterparts of SARS-CoV, but not other human β-CoVs.

### NTD- and CTD- Ab responses elicited during SARS-CoV-2 natural infection

**Figure 4:**
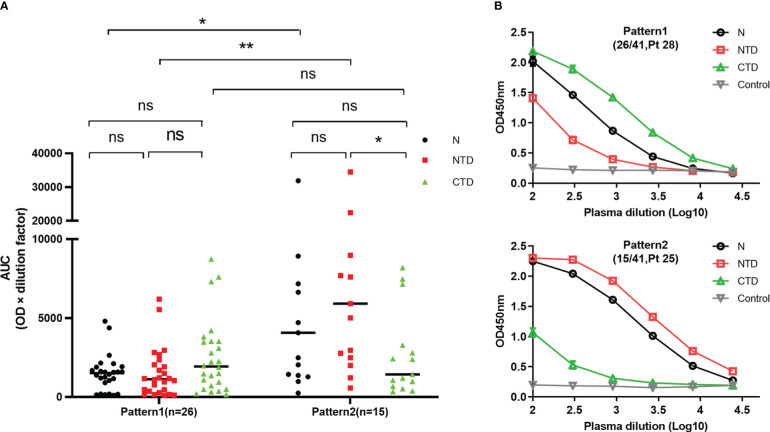
Patterns of COVID–19 patient plasma Ab responses to SARS–CoV–2 N protein. **(A)** ELISA results showing the patterns of COVID–19 patient plasma Ab responses to SARS–CoV–2 N protein. In Pattern1, Abs in 26 plasma samples reacted with NTD, CTD, and full–length N proteins at comparable levels, While in pattern2, NTD–Abs were dominant in 15 plasma samples. **(B)** The binding curves of Pattern1 and Pattern2 to NTD, CTD, and full–length N proteins, represented by the tested plasma samples, Pt 28 and Pt 25, respectively. The plasma samples were diluted at 1:100 dilution and followed by 3–fold serial dilution. Diluted plasma samples were subjected to ELISA to measure anti–N protein Abs. Each dilution was tested in duplicate. AUC was calculated using GraphPad Prism 8. N, full–length; NTD, N–terminal domain; CTD, C–terminal domain; ns, not significant; Control, supernatant from 293 T cells without transfection; *p< 0.05, **p< 0.01.

We also studied whether Abs against the N protein NTD and CTD were elicited during SARS-CoV-2 natural infection. A total of 52 plasma samples from convalescent COVID-19 patients were used for ELISA analysis of Ab responses to the full-length and truncated fragments of the N proteins. Forty-one (78.8%) plasma samples had detectable levels of N-specific Abs. According to the binding pattern of each sample to NTD and CTD, two different responsive patterns were observed, namely Pattern1 and Pattern2 (**Figure 4**; **Supplementary Table 1**). In Pattern1, Abs in 26 plasma samples reacted with NTD, CTD, and full-length N proteins at comparable levels, although CTD-Ab level appeared slightly higher than that of total N-Abs (*p*=0.0525) (**Figure 4**). While in pattern2, NTD-Abs were dominant in 15 plasma samples (*p*=0.026, NTD-Abs vs CTD-Abs in pattern2). The levels of N-Abs and NTD-Abs in pattern2 were higher than in pattern1 (*p*=0.03 and *p*=0.009, respectively, for their comparisons between pattern1 and pattern2) (**Figure 4**). The levels of CTD-Abs in these two patterns were comparable (*p*=0.8375) (**Figure 4**). These results indicate that Abs against the NTD and CTD of SARS-CoV-2 N protein are elicited during natural infection and that dominant anti-NTD Ab responses occur in some infected individuals.

### The cross-reactivities of N-specific Abs in COVID-19 patients with other human β-CoVs

**Figure 5:**
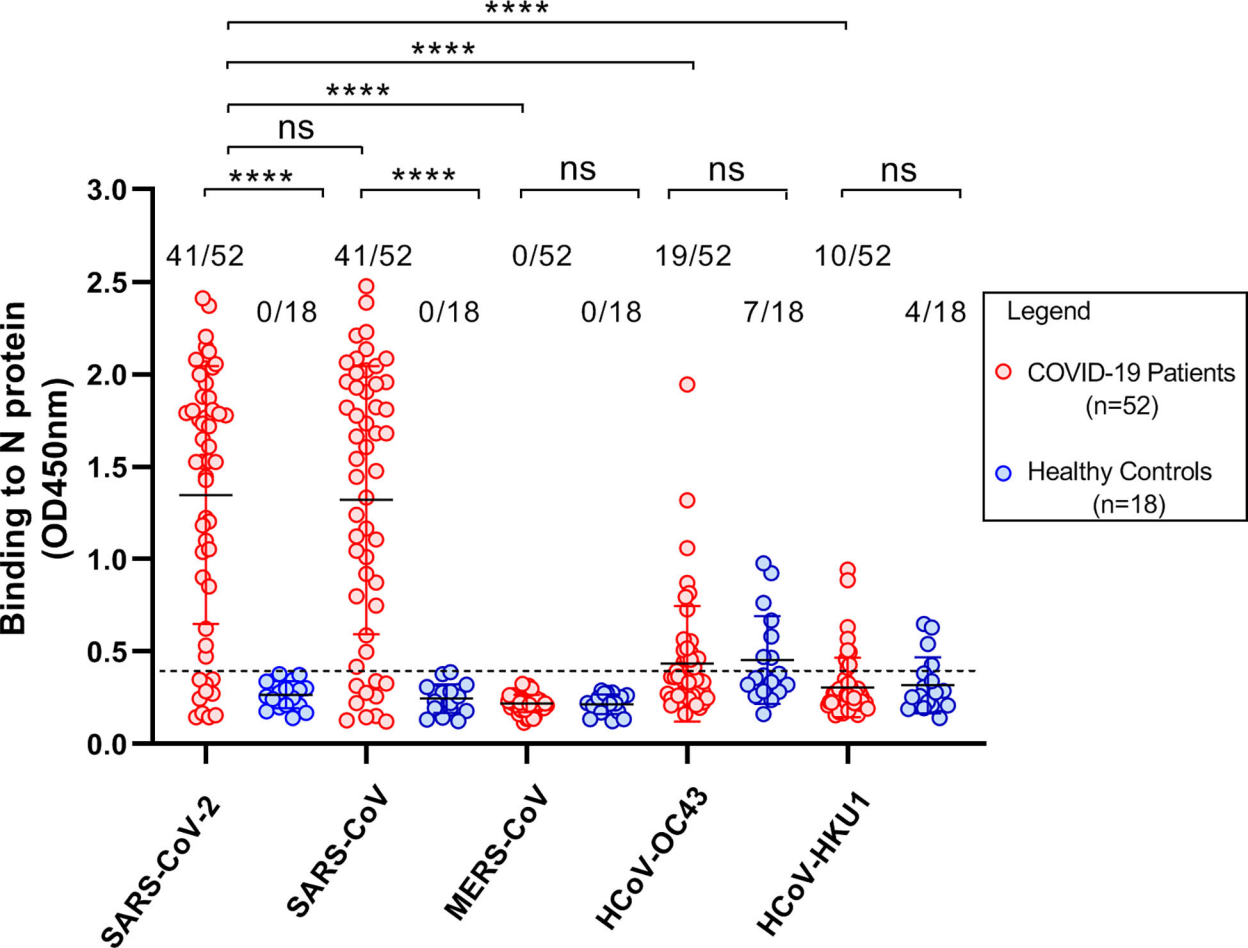
Cross–reactivity of Abs in plasma samples to the N proteins of human β–CoVs. Scatter plots demonstrating ELISA results that showed the cross–reactivity of Abs in plasma samples from COVID–19 patients and healthy blood donors to the N proteins of human β–CoVs including SARS–CoV–2, SARS –CoV, MERS–CoV, OC43, and HKU1. The plasma samples diluted at 1:100 were used. All samples were detected in duplicate. Eighteen plasma samples from healthy blood donors were collected before the COVID–19 pandemic. All comparisons were conducted using unpaired *t* test *via* GraphPad Prism 8. A p–value less than 0.05 (*p*< 0.05) was statistically significant. ns, not significant; ****p< 0.0001.

Recent studies have demonstrated that convalescent COVID-19 sera contain high levels of cross-reactive Abs to SARS-CoV structural proteins indicating robust cross-reactive Abs are elicited during natural SARS-CoV-2 infection (28–30). To confirm these data and also to clarify whether N-specific Abs in COVID-19 patients cross-reacted with N proteins of other human β-CoVs, we first screened Abs in plasma samples that were collected from 18 healthy blood donors to the N proteins of all five human β-CoVs including SARS-CoV-2, SARS-CoV, MERS-CoV, OC43, and HKU1. Abs to OC43 and HKU1 N proteins were detected in 38.9% (7 out of 18) and 22.2% (4 out of 18) of plasma samples, respectively (**Figure 5**). There was no plasma sample that had detectable Abs to the N proteins of SARS-CoV-2, SARS-CoV, or MERS-CoV(**Figure 5**; **Supplementary Figure 5** and **Supplementary Table 2**). Thus, prior to the COVID-19 pandemic, approximately 22 - 39% healthy individuals have detectable Abs against the N proteins of OC43 and HKU1, the causative agents of common cold, while none of them have detectable Abs to the N proteins of SARS-CoV-2, SARS-CoV, or MERS-CoV.

Next, we tested 52 plasma samples collected from COVID-19 patients for Abs against the N proteins of SARS-CoV-2, SARS-CoV, MERS-CoV, OC43, and HKU1. As described above, Abs against SARS-CoV-2 N protein were detected in 78.8% (41 out of 52) of plasma samples from these COVID-19 patients. We also found that these 41 plasma samples had cross-reactive Abs to the N protein of SARS-CoV. The average OD values were 1.35 ± 0.70 and 1.32 ± 0.72 for the N proteins of SARS-CoV-2 and SARS-CoV, respectively (**Figure 5**), suggesting that the cross-reactive Abs react equally with the N proteins of SARS-CoV-2 and SARS-CoV. No plasma sample had cross-reactive Abs to the N protein of MERS-CoV (**Figure 5**; **Supplementary Figure 5**). Cross-reactive Abs to the N proteins of OC43 and HKU1 were detected in 36.5% (19/52) and 19.2% (10/52) of samples, respectively (**Figure 5**). These detection frequencies are similar to those observed in healthy blood donors (**Figure 5**; **Supplementary Table 2**).

## Discussion

In this study, five mAbs against SARS-CoV-2 N protein were isolated from memory B cells in the peripheral blood of two individuals who had recovered from COVID-19. Epitope mapping revealed that three of these human mAbs (N3, N5, and N31) were reactive with the CTD, while the other two mAbs (N83 and 3B7) targeted the NTD of SARS-CoV-2 N protein. All five patient-derived mAbs to SAR-CoV-2 N protein were cross-reactive to the N protein of SARS-CoV but showed little to no cross-reactivity to the N proteins of other human β-CoVs including MERS-CoV, OC43, and HKU1. These results are likely reflected by the amino acid sequence similarities of the N proteins from these human β-CoVs. Among them SARS-CoV-2 and SARS-CoV N proteins are highly similar in their amino acid sequences and likely share more common antigenic epitopes than other human β-CoVs (20–23).The similarities in the NTD and CTD amino acid sequences of SARS-CoV-2 and SARS-CoV are particularly high, having 92.2% and 95.8% identity, respectively (**Supplementary Figure 6**; **Supplementary Table 3**). Such high similarities in the NTD and CTD amino acid sequences of SARS-CoV-2 and SARS-CoV are expected to elicit cross-reactive Abs as we demonstrated above. Notably, the five patient-derived hmAbs in our study were all IgG isotypes. A recent study has demonstrated that one mouse-derived IgM mAb against SARS-CoV N protein can strongly cross-react with SARS-CoV-2 N protein (24), although the antigenic epitope of this mouse IgM mAb has not been determined. Taken together, the N proteins of SARS-CoV-2 and SARS-CoV, particularly their NTD and CTD domains, have high identity and similarity and share some common antigenic epitopes that elicit cross-reactive Abs of various immunoglobulin classes.

We also used the full-length and truncated fragments of SARS-CoV-2 N protein to characterize antigenic domains of anti-N Abs that were elicited during SARS-CoV-2 natural infection. We found that 78.8% (41/52) of plasma samples had detectable levels of N-specific Abs. Among these plasma samples with detectable N-specific Abs, Abs against NTD and CTD of SARS-CoV-2 N protein are elicited during natural infection and dominant anti-NTD Ab responses occur in some infected individuals. These Abs against NTD and CTD of SARS-CoV-2 N protein do not have neutralizing activity, their contributions to the antiviral response need to be studied further. Growing evidence has indicated that N-specific T cell immunity plays a critical role in protection against SARS-CoV-2 infection (41–46), and that SARS-CoV-2 vaccines could be improved by incorporating the N protein as an antigen (47). Indeed, inclusion of SARS-CoV-2 N protein in future COVID-19 vaccines and boosters has emerged as a promising strategy (48–50). SARS-CoV-2 N protein is more conservative and less mutated in comparison to the S protein, making it an ideal component of novel COVID-19 vaccines to prevent immune escape by SARS-CoV-2 variants.

Currently, N-specific Abs used for N antigen detection are solely derived from animals such as mice (51–54). Because of differences in immune background and responses to immunization, mouse–derived Abs against the SARS–CoV–2 N protein may not sufficiently define antigenic epitopes of N protein during natural SARS–CoV–2 infection. Indeed, one study has shown a distinct trend towards mouse–derived N–specific mAbs preferentially targeting the NTD of the N protein (51). Our patient–derived mAbs were cross–reactive to the N protein of SARS–CoV, but showed little to no cross–reactivity to the N proteins of other human β–CoVs. Since our mAbs were directly derived from B cells in the peripheral blood of COVID–19 patients, epitope mapping with these patient–derived mAbs likely reflects real–world conditions better than animal–derived mAbs and may be more precise for mapping antigenic determinants and B cell epitopes as well. Given that these patient–derived mAbs specifically recognized the N proteins of SARS–CoV–2 and SARS–CoV and that only a small number of people (8,422 confirmed cases) worldwide were infected by SARS–CoV during the 2003 outbreak (55), they can be potentially used for developing the next generation of COVID–19 At–Home Test kits for a rapid and specific screening of SARS–CoV–2 infection and EIAs for clinical laboratory diagnosis of COVID–19.

In summary, five patient–derived mAbs to SARS–CoV–2 N protein were isolated from memory B cells in the peripheral blood of convalescent COVID–19 patients. These mAbs targeted the CTD and NTD of SARS–CoV–2 N protein and were cross–reactive to the N protein of SARS–CoV, but not other human β–CoVs. These mAbs can be potentially used for developing the next generation of COVID–19 At–Home Test kits for a rapid and specific screening of SARS–CoV–2 infection. Our results also provide insights into the development of novel COVID–19 vaccines to fight SARS–CoV–2 variants.

## Data availability statement

The original contributions presented in the study are included in the article/**Supplementary Material**. Further inquiries can be directed to the corresponding author.

## Ethics statement

The studies involving human participants were reviewed and approved by the Institutional Review Boards (IRB) at the Guangzhou Eighth People’s Hospital (20200134). The patients/participants provided their written informed consent to participate in this study.

## Author contributions

LY conceived the research project, designed the study, interpreted data, directed the entire research activities, and wrote the manuscript. YW, WG, KZ, XZ and XX performed experiments. YM, YT, YP, WH, and WC managed patients and collected clinical samples. All authors contributed to the article and approved the submitted version.
