# Supplementary material for: Patient-derived monoclonal antibodies to SARS-CoV-2 nucleocapsid protein N-terminal and C-terminal domains cross-react with their counterparts of SARS-CoV, but not other human betacoronaviruses
Source: Front Immunol. 2023 Jan 31;14:1093709. doi: 10.3389/fimmu.2023.1093709 (PMC9927002; doi:10.3389/fimmu.2023.1093709)
Supplement: Supplementary file 1 [file DataSheet_1.docx]

**Supplementary Data**

**Patient-derived monoclonal antibodies to SARS-CoV-2 nucleocapsid protein N-terminal and C-terminal domains cross-react with their counterparts of SARS-CoV, but not other human betacoronaviruses**

Yingfen Wen^*^, Wenjing Guo^*^, Yuyi Min, Kexin Zhong, Xulei Zhang, Xiaomin Xing, Yuwei Tong, Yuejun Pan, Wenxin Hong, Weiping Cai, Lei Yu^€^

Guangzhou Eighth People’s Hospital, Guangzhou Medical University, Guangzhou 510060, China

^€^Co-corresponding authors.

Address correspondence to Dr. Lei Yu ([leiyu11393@126.com](mailto:leiyu11393@126.com)), Guangzhou Eighth People’s Hospital, Guangzhou Medical University, 627 Dongfeng Rd. East, Guangzhou 510060, China.

*Y.W. and W.G. equally contributed to this work

**Key words:** SARS-CoV-2, coronavirus, COVID-19, nucleocapsid protein, N-terminal, C-terminal, monoclonal antibody, cross-reaction

**Running title:** Cross-reactivity of SARS-CoV-2 N-specific antibodies

**Supplementary Figure**

**
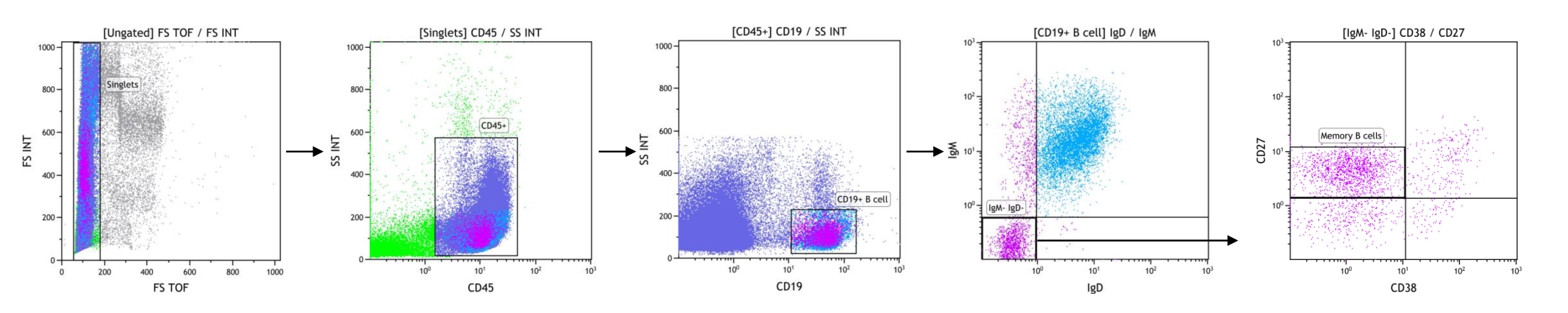
Supplementary Figure 1. The gating strategy for memory B cell Sorting.** The first gate was on singlets based on FS TOF vs FS INT. After gating on CD45, then CD19, IgD-IgM- B cells were identified using IgD and IgM expression. IgD-IgM- B cells were further analyzed by CD38 and CD27 expression. CD19^+^IgD^-^IgM^-^CD27^+^CD38^low^ cells were identified as memory B cells.

**
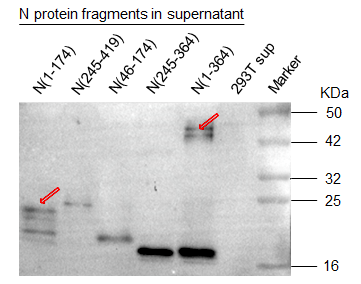
**

**Supplementary Figure 2. Expression of SARS-CoV-2 N protein fragments.** SDS-PAGE electrophoresis showing a panel of overlapping N protein fragments of SARS-CoV-2 including N(1-174), N(245-419),N(46-174), N(245-364) and N(1-364) in the supernatant of transfected 293 T cells. Supernatant from 293 T cells transfected with empty pcDNA3.1 vector was used as a control (293T sup).


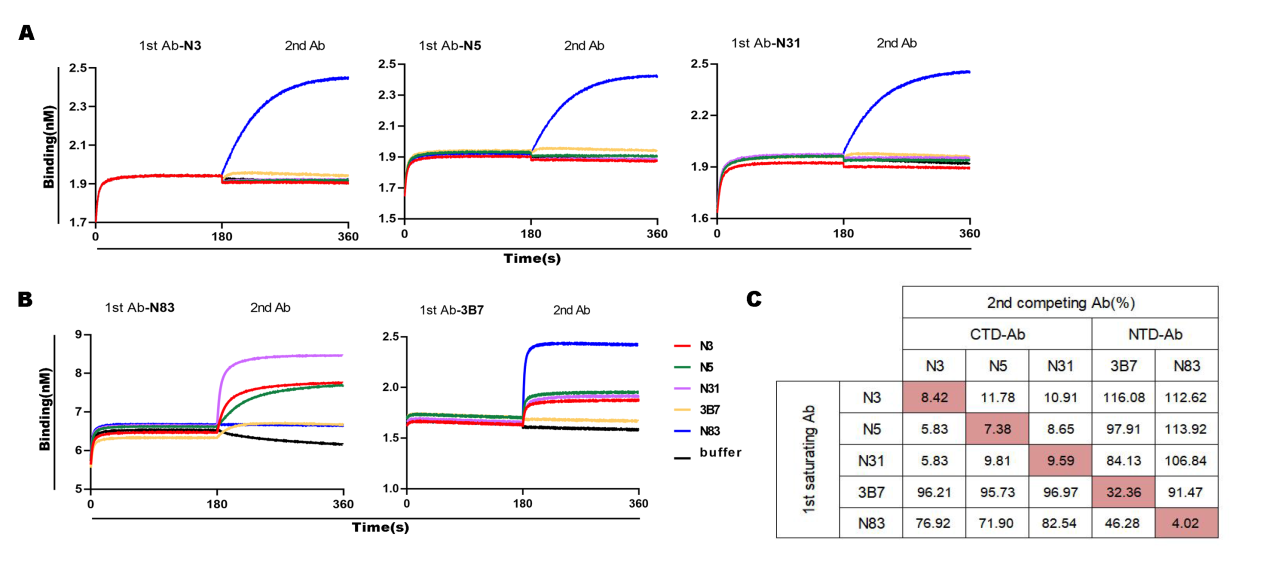


**Supplementary Figure 3. The epitope binning of SARS-CoV-2 N-specific hmAbs detected by BLI.** The SARS-CoV-2 N protein was captured by anti-tag antibody with biotin labeling, which was immobilized on SA sensor. The first saturating hmAb was added and followed by the second competing hmAb **(A-B)**. The percentage of residual binding was calculated as binding value of second antibody in the presence of first antibody divided by the binding value of second antibody alone **(C)**.

**
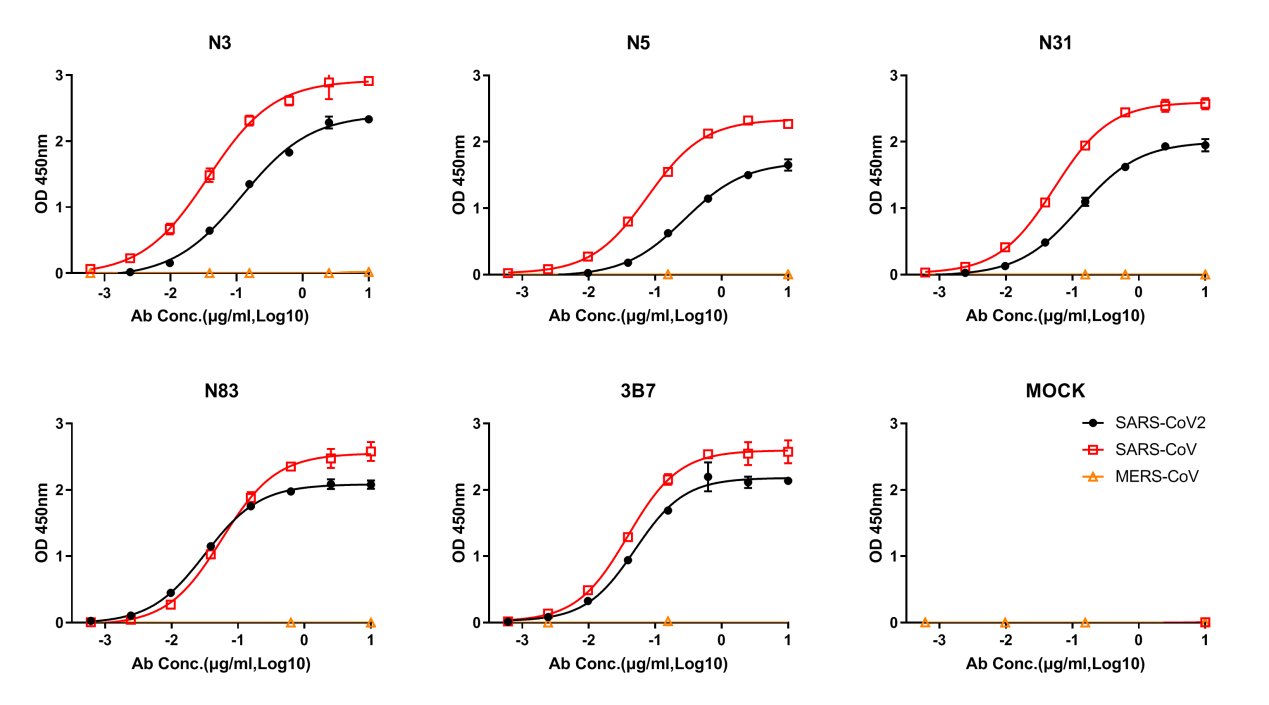
Supplementary Figure 4. Patient-derived mAbs cross-reacted with the N proteins of human β-CoVs.** ELISA results showing the binding curves of Abs to the commercial N proteins of SARS-CoV-2, SARS-CoV, and MERS-CoV (Sino Biological, Inc., China). MOCK, one hmAb (3G2) specifically binding to NS1 protein of Zika virus.

**
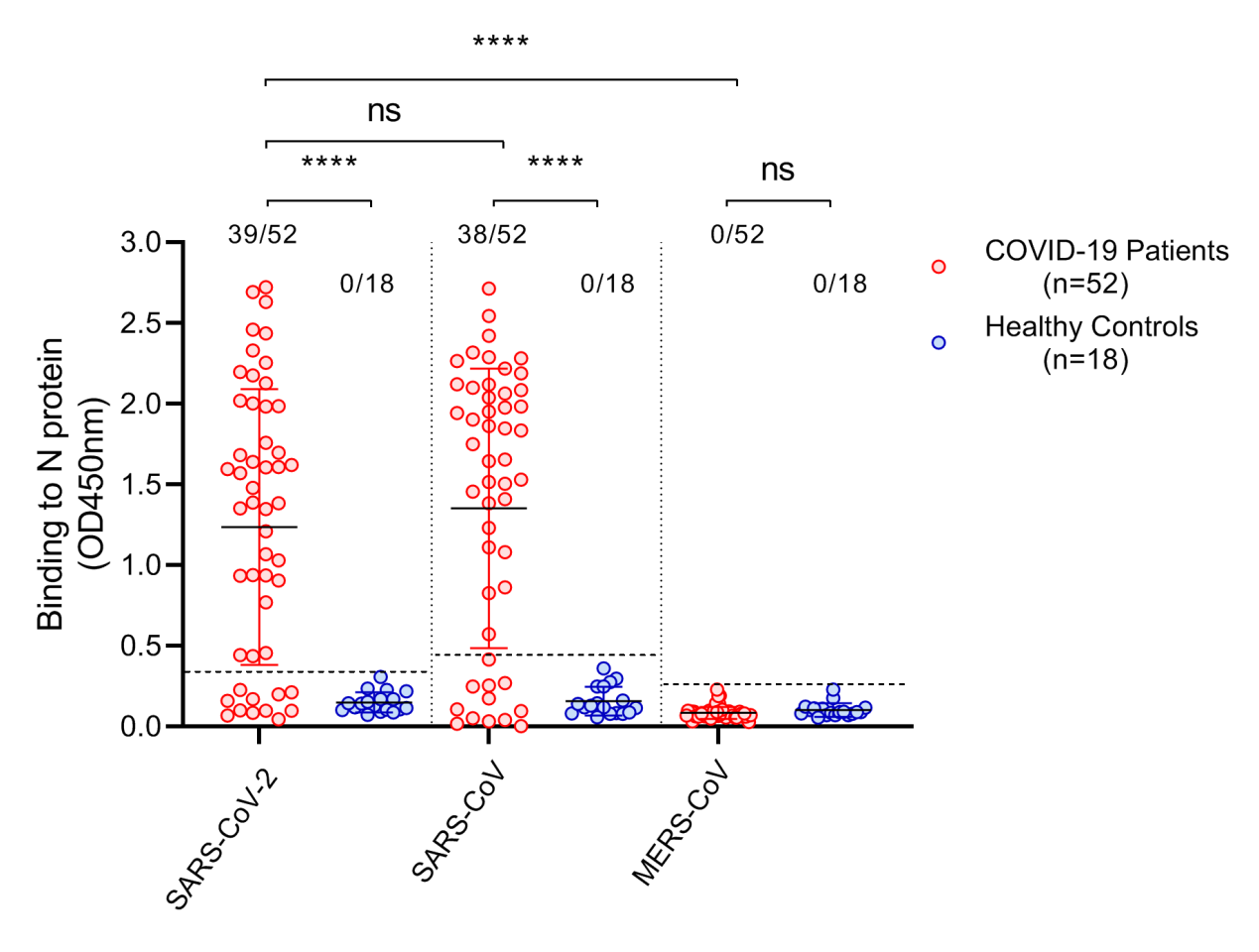
Supplementary Figure 5. Cross-reactivity of Abs in plasma samples to the N proteins of human β-CoVs.** Scatter plots demonstrating ELISA results that showed the cross-reactivity of Abs in plasma samples from COVID-19 patients and healthy blood donors to the commercial N proteins of SARS-CoV-2, SARS-CoV, and MERS-CoV (Sino Biological, Inc., China). The plasma samples diluted at 1:100 were used. All samples were detected in duplicate. Eighteen plasma samples from healthy blood donors were collected before the COVID-19 pandemic. All comparisons were conducted using unpaired *t* test via GraphPad Prism 8. A p-value less than 0.05 (*p*<0.05) was statistically significant. ns, not significant; *****p*<0.0001.


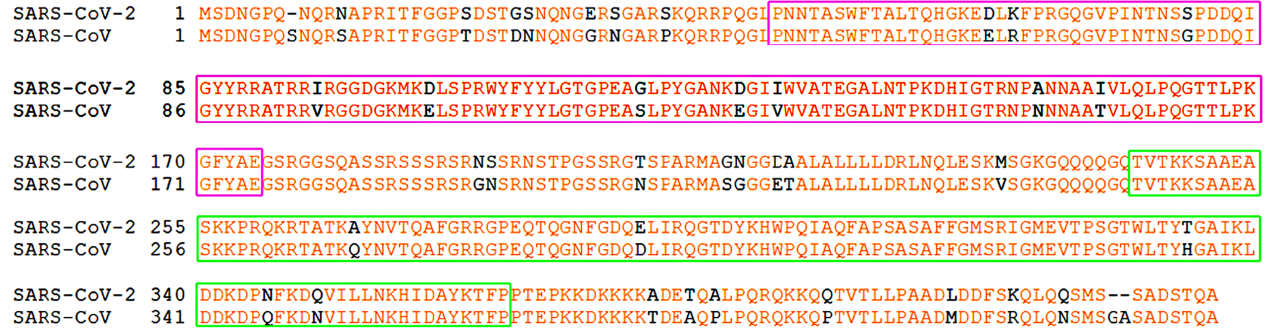


**Supplementary Figure 6. The sequence alignment of SARS-CoV-2 and SARS-CoV N proteins.** The similarity of SARS-Cov-2 and SARS-CoV N proteins is high, 90.5% for full length, 92.2% for NTD domain (Pink highlighted region) and 95.8% for CTD domain (Green highlighted region).

**Supplementary Table1** Demographics and clinical information of recovered COVID-19 patients (n=41) with detectable N-specific Abs.

|  | Pattern1 (n=26) | Pattern2 (n=15) | *P* value |
| --- | --- | --- | --- |
|  |  |  |  |
| Female sex, no. (%) | 14(54%) | 6(40%) | 0.393^a^ |
| Median age in years (range) | 43.5(18-77) | 47(29-68) | 0.291^b^ |
|  |  |  |  |
| **Clinical type, no. (%)*** | 23 | 14 |  |
| Mild | 4(17%) | 1(7%) | 0.203^c^ |
| Moderate | 19(83%) | 12(86%) |  |
| Severe | 0(0%) | 1(7%) |  |
| a chi-square test. |  |  |  |
| b Unpaired t test with Welch's correction | |  |  |
| c Mann Whitney U test |  |  |  |

*****The clinical information of four individuals are not available, three in Pattern1, one in Pattern2.

**Supplementary Table2** N-specific antibodies of SARS-CoV-2, SARS-CoV, MERS-CoV, OC43, and HKU1 in 18 healthy blood donors detected by ELISA.

| Sample # | SARS-CoV-2 | SARS-CoV | MERS-CoV | HCoV-OC43 | HCoV-HKU1 |
| --- | --- | --- | --- | --- | --- |
| Ab-positive donors | 0/18 | 0/18 | 0/18 | 7/18 | 4/18 |
| 17 | 0.3778 | 0.3877 | 0.2181 | 0.6686 | 0.5401 |
| 10 | 0.2478 | 0.2219 | 0.1749 | 0.9780 | 0.6294 |
| 8 | 0.2580 | 0.2414 | 0.2204 | 0.9249 | 0.6486 |
| 5 | 0.3014 | 0.2783 | 0.2533 | 0.7624 | 0.4270 |
|  |  |  |  |  |  |
| 14 | 0.2416 | 0.2302 | 0.2060 | 0.5815 | 0.2527 |
| 6 | 0.3454 | 0.3209 | 0.2891 | 0.4676 | 0.3824 |
| 16 | 0.3384 | 0.3075 | 0.2747 | 0.4713 | 0.3406 |
|  |  |  |  |  |  |
| 2 | 0.2078 | 0.1787 | 0.1933 | 0.3721 | 0.2135 |
| 4 | 0.3399 | 0.2866 | 0.2626 | 0.3805 | 0.2878 |
| 7 | 0.3734 | 0.3782 | 0.2774 | 0.3563 | 0.3084 |
| 9 | 0.2939 | 0.2779 | 0.2754 | 0.3216 | 0.2593 |
| 11 | 0.1759 | 0.1432 | 0.1344 | 0.2819 | 0.1902 |
| 13 | 0.2794 | 0.2566 | 0.2199 | 0.2389 | 0.2268 |
| 15 | 0.2966 | 0.2833 | 0.2491 | 0.3346 | 0.2774 |
| 18 | 0.2101 | 0.1932 | 0.1713 | 0.2847 | 0.2080 |
| 19 | 0.1683 | 0.1323 | 0.1336 | 0.3215 | 0.1939 |
| 1 | 0.1981 | 0.1912 | 0.1973 | 0.2586 | 0.2085 |
|  |  |  |  |  |  |
| 3 | 0.1406 | 0.1245 | 0.1245 | 0.1623 | 0.1400 |

The threshold of OD value for Ab-positive was calculated by the mean value of negative controls plus 3X standard deviation.

**Supplementary Table3** The sequence similarity (%) of N protein of SARS-CoV-2 to other human CoVs .

| **SARS-CoV-2 N protein (Amino acids)** | | **N**  (419) | **NTD** (129) | **CTD** (120) |
| --- | --- | --- | --- | --- |
| **Beta-CoV** | SARS-CoV | 90.5 | 92.2 | 95.8 |
|  | MERS-CoV | 45.1 | 59.7 | 51.6 |
|  | HCoV-OC43 | 31.5 | 41.2 | 32.8 |
|  | HCoV-HKU1 | 29.1 | 42.2 | 35.6 |
| **Alpha-CoV** | HCoV-229E | 22.9 | 27.5 | 25.8 |
